# Comparison of single CT scan assessment of bone mineral density, vascular calcification and fat mass with standard clinical measurements in renal transplant subjects: the ABC HeART study

**DOI:** 10.1186/s12882-015-0182-6

**Published:** 2015-11-11

**Authors:** Sinead Kinsella, Kevin Murphy, Micheal Breen, Siobhan O’Neill, Patrick McLaughlin, Joe Coyle, Conor Bogue, Fiona O’Neill, Niamh Moore, AnneMarie McGarrigle, Michael G. Molloy, Michael M. Maher, Joseph A. Eustace

**Affiliations:** Department of Renal Medicine, Cork University Hospital, Cork, Ireland; Department of Radiology, Cork University Hospital, Cork, Ireland; Department of Physics, Cork University Hospital, Cork, Ireland; Department of Rheumatology, Cork University Hospital, Cork, Ireland; HRB Clinical Research Facility at UCC, 2nd Floor, Mercy University Hospital, Grenville Place, Cork, Ireland

**Keywords:** Renal transplantation, Bone mineral density, Vascular calcification, Adiposity, Cardiovascular disease, Risk factors

## Abstract

**Background:**

Despite limitations of routine methods, Clinical Practice Guidelines support the assessment of bone mineral density (BMD) and vascular calcification in renal transplant recipients. Changes in fat mass also occur post-transplantation, although they are traditionally difficult to measure accurately. We report the feasibility, convenience and accuracy of measuring the above 3 parameters using a novel CT protocol.

**Methods:**

We conducted a cross-sectional study of 64 first renal allograft recipients (eGFR > 30 ml/min/1.73 m^2^). Quantitative CT (QCT) BMD analysis was conducted using CT lumbar spine (GE Medical Systems Lightspeed VCT & Mindways QCT Pro Bone Mineral Densitometry System Version 4.2.3) to calculate spinal volumetric BMD and compared with standard DXA calculated areal BMD at the spine, hip and distal forearm. Abdominal aortic calcification was assessed by semi-quantitative Aortic Calcification Index (ACI) method and compared with lateral lumbar x-ray Kappuila score and pulse wave velocity (PWV). Visceral and subcutaneous adipose tissue volume (Osirix 16 Ver 3.7.1) was compared with BMI.

**Results:**

Participants were 61 % male, had a mean age of 47 years, median ESKD duration of 5.4 years and a mean eGFR of 54 ml/min. iDXA median T-score at proximal femur was −1.2 and at lumbar spine was −0.2. Median QCT Trabecular T-score at lumbar spine was −1.2. The percent of subjects with a T-score of <2.5 by site and method was DXA Proximal Femur: 7 %, DXA distal radius: 17 %, DXA spine: 9 %, QCT (American College of Radiology cutoffs): 9 %. CT derived ACI correlated with PWV (*r* = 0.29, *p* = 0.02), pulse wave pressure (*r* = 0.51, *p* < 0.001), QCT Trabecular (−0.31, *p* = 0.01) and cortical volumetric BMD and history of cardiovascular events (Mann–Whitney U, *p* = 0.02). Both visceral and subcutaneous adipose tissue correlated with BMI (*r* = 0.63 & 0.64, *p* < 0.001).

**Conclusions:**

Single CT scan triple assessment of BMD, vascular calcification and body composition is an efficient, accurate and convenient method of risk factor monitoring post renal transplantation.

## Background

Renal transplantation restores endogenous renal function in subjects with End Stage Kidney Disease and is the treatment of choice for suitable patients with renal failure. However, cardiovascular mortality and fragility fracture risk remain chronically elevated post renal transplantation and persisting disturbances in bone mineral metabolism are implicated in the genesis of both of these complications.

The National Kidney Foundation ‘Kidney Disease Outcomes Quality Initiative’ and other guidelines recommend utilising dual energy x-ray absorptiometry (DXA) at various intervals post renal transplantation to monitor bone mineral density [1, 2]. A well-recognized limitation of this technique of particular concern in renal failure is the overestimation of lumbar BMD due to the presence of extraosseous calcification especially overlying aortic calcification. Alternative methods of assessing bone mineral density, such as quantitative CT (QCT) are therefore particularly appealing in renal transplant recipients and have the added advantage of distinguishing between cortical –which is preferentially influenced by elevated parathyroid hormone- and trabecular Bone Mineral Density. The utility of both QCT has been explored in dialysis dependent populations [3–5] but not in the post renal transplant setting.

In both the general and renal populations vascular calcification is believed to be a risk marker of cardiovascular morbidity [6]. A number of non-invasive methods are available to assess vascular calcification and the resulting arterial stiffness including plain radiography, CT assessment of arterial calcification and arterial pulse wave velocity. Lateral Bone Densitometry has also been used to assess vertebral BMD and detect vascular calcification in hemodialysis [7] and CKD [8] patients. The KDIGO Clinical Practice Guideline group in 2006 recommended that lateral abdominal radiography be used as a screening tool for the detection of vascular calcification in patients with CKD Stages 3-5D [9]. A specific guideline does not exist for the assessment of vascular calcification in the post-transplant group but surveillance is recommended [10].

While plain radiographs provide a semi-quantitative anatomical assessment of the presence of vascular calcification and Pulse Wave Velocity provides a functional assessment of the resultant aortic stiffness, Computed Tomography provides a more accurate method of quantifying the burden of vascular calcification. Their use is much enhanced by the now widespread availability of new generation CT scanners and newer software systems that can facilitate low radiation dose protocols with acceptable diagnostic accuracy.

An additional area of increasing research and clinical interest is the assessment of the change in fat mass post transplantation and its resulting clinical consequences. This results in part from the use of immunosuppressive medications particularly corticosteroids and is potentially associated with insulin resistance, new onset diabetes after transplantation and “diabesity” associated metabolic syndrome and cardiovascular morbidity [11, 12]. Changes in visceral adipose tissue are believed to be more relevant than changes in subcutaneous adipose tissue but accurate assessment of visceral fat mass is challenging and difficult to standardize in routine clinical practice but can be reliably assessed by CT [13–15].

As part of an ongoing prospective cohort study quantifying bone and vascular risk factors in successful renal transplant recipients (The ABC HeART study) we compared the use of a specifically designed CT protocol, designed to facilitate contemporaneous triple assessment of lumbar-sacral BMD, aortic calcification and visceral fat mass with routine clinical measurements.

## Methods

ABC HeART is a single centre cohort study of renal transplant recipients. To be eligible subjects had to be between 0.5 and 12 years post engraftment of their first renal transplant, to have an enrollment eGFR > 30 ml/min/1.73 m^2^ and to be in their usual state of health. All study procedures were performed on an outpatient basis. Informed written consent was obtained from all participants following approval of the study protocol by the Clinical Research Ethics Committee of the Cork Teaching Hospitals. Subject demography, clinical details and past medical, fracture and cardiovascular history were obtained by patient self-report and abstracted from their medical records. Weight and height were measured to calculate Body Mass Index.

Areal Bone Mineral Density was measured at the lumbar spine, the femoral neck and proximal femur of both native hips and the non-dominant forearm, using a Lunar IDXA scanner (General Electric) and expressed as both areal Bone Mineral Density and as a T-score. In keeping with the World Health Organization (WHO) definition, osteoporosis was defined as a lowest T-score of −2.5 or less and osteopenia as a T-score of −1.0 to −2.49, using the Proximal Femur as the reference standard.

Carotid Femoral Pulse Wave Velocity was performed by a single investigator (SK) experienced in the performance of the technique using a dedicated Pulse Trace 400 PWV system (Viasys Healthcare) following the method described by London [16].

Presence of aortic calcification was assessed by 2 radiologists who were blinded to the clinical study detail. Aortic vascular calcification was quantified from lateral lumbar spine radiographs using the Framingham method, scoring on a scale of 1–3 the presence of calcification along the anterior and posterior margin of the expected aortic contour adjacent to the 4 lumbar vertebrae, resulting in a composite score of between 0–24 [17].

To facilitate contemporaneous triple assessment of lumbar BMD, aortic calcification and visceral fat mass a specifically designed CT protocol was developed. A 64 slice General Electric Medical Systems Lightspeed VCT XTE scanner was used for evaluation. A helical series from the L1 to L4 vertebral bodies (modulated 20 to 80 mAs, NI 28, kVp 120) was performed in all patients. An acquisition slice thickness of 0.625 mm was utilised with a pitch of 0.984:1, a noise index of 28 (modulated) and a large field of view. Regular calibration using the BMD phantom was performed as per manufacturer guidelines. Bone Mineral Density, aortic calcification and visceral and subcutaneous fat mass was determined on each CT scan by 2 radiologists who were blinded to all study data.

**Fig. 1:**
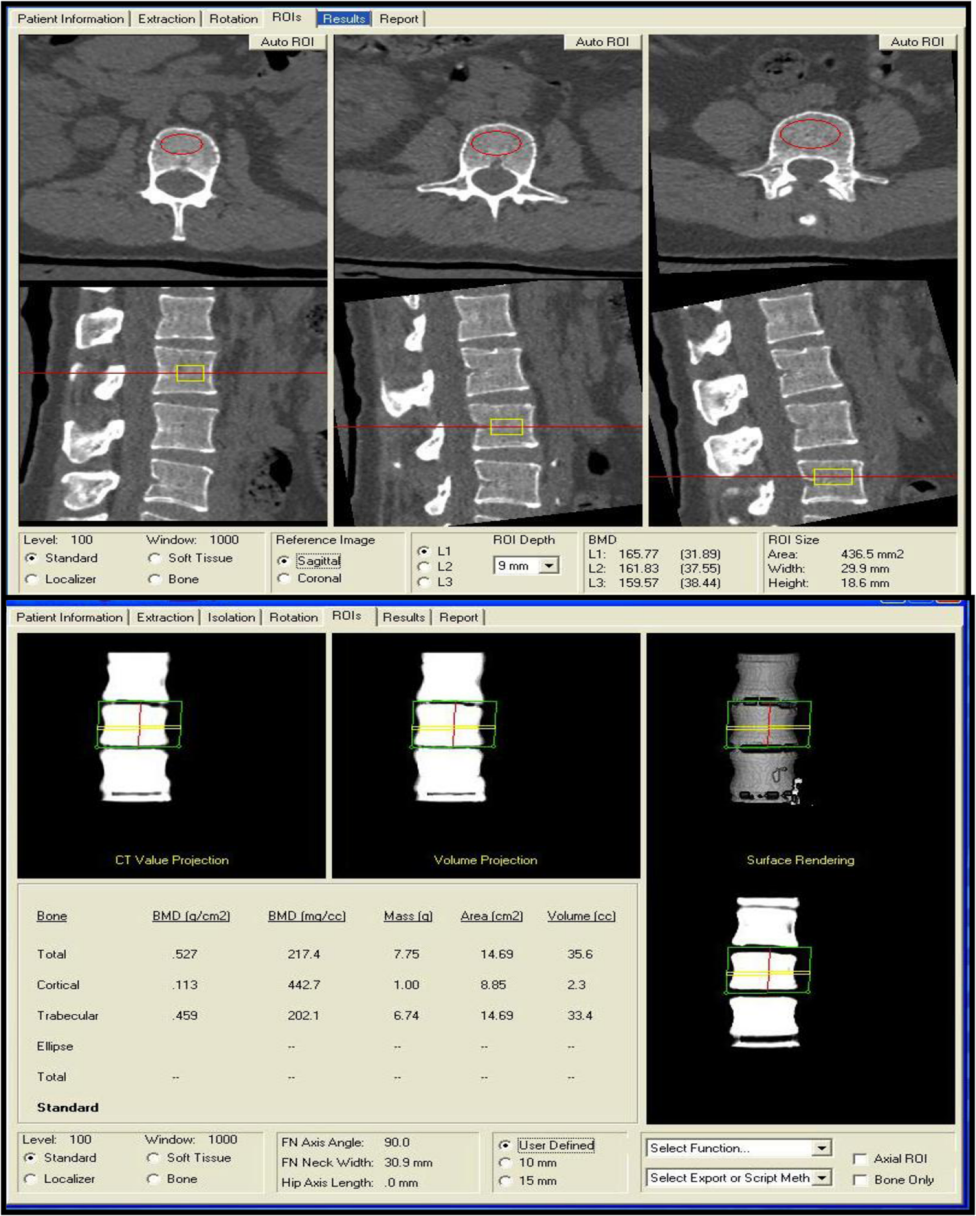
Trabecular and Cortical QCT BMD calculation using the Mindways QCT Pro Bone Mineral Densitometry System Version 4.2.3

Volumetric cortical and trabecular bone mineral density in g/cm^3^ of the L1 to L3 vertebral bodies was analysed using the Mindways QCT Pro Bone Mineral Densitometry System Version 4.2.3, Fig. 1. For trabecular BMD this also calculates a technique-specific vertebral T score, expressing the measured BMD in terms of the number of standard deviations above or below a gender specific young adult reference population. However, while this T-score is informative regarding spinal BMD relative to peak bone mass seen in young adults, it is neither identical to nor interchangeable with the areal BMD T score measured at the proximal femur, as is used as the reference standard in the WHO definition of osteoporosis and in the FRAX calculation of estimated long term fracture risk. The American College of Radiology has recently revised its ‘Practise Parameter for the performance of Quantitative Computed Tomography (QCT) Bone Densitometry’ (http://www.acr.org/~/media/DE78D218C7A64526A821A9E8645AB46D.pdf, accessed 5/09/2015) which provides recommendations on the interpretation of spinal QCT in the absence of femoral measurements. The revised Practise Parameter recommends using trabecular QCT volumetric BMD cut-offs of <120 g/cm^3^ and <80 g/cm^3^ to estimate the relevant diagnostic thresholds for osteopenia and osteopenia that equate to those based on proximal femoral areal BMD measurements. Therefore in addition to the actual measurements of volumetric BMD, in the case of trabecular bone we also report both the QCT derived vertebral T-score and the proportion of subjects meeting the above mentioned American College of Radiology diagnostic cut-offs.

**Fig. 2:**
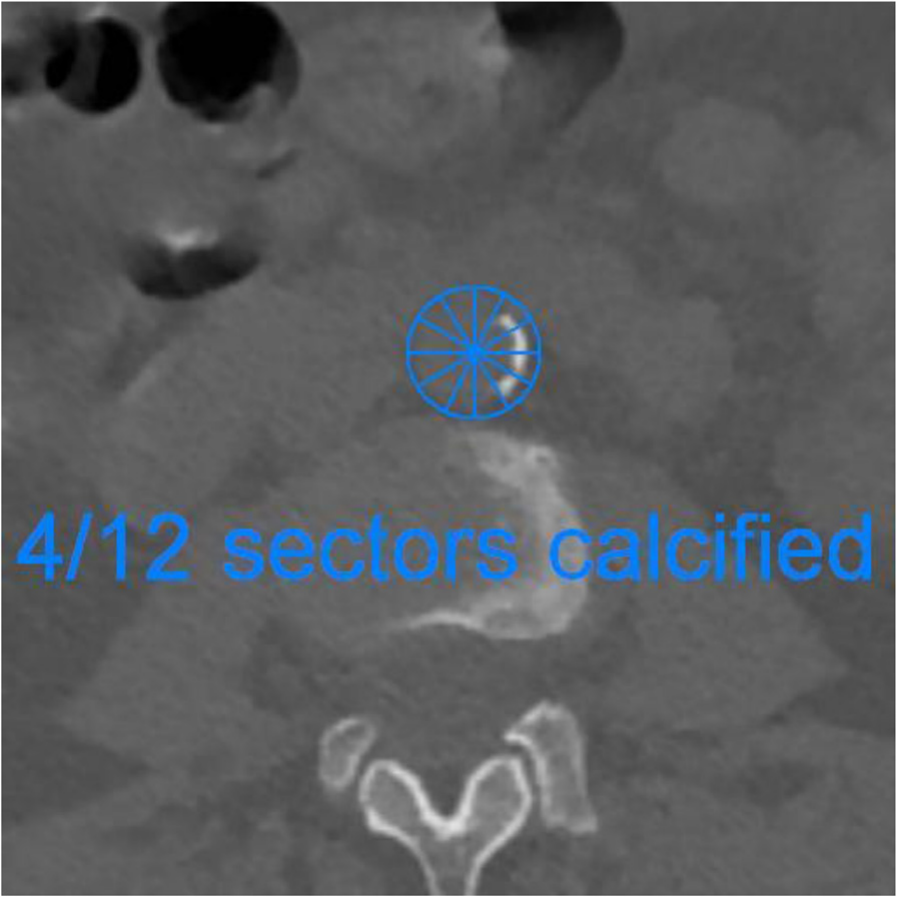
Calcification assessment using the aortic calcification index (ACI)

The extent of arterial calcification in the abdominal aorta detected by CT was evaluated using 2 standardized methods [18–20]; a volumetric analysis using threshold based segmentation (Advantage workstation, GE) and a semi-quantitative Aortic Calcification Index (ACI) which involves visual assessment of 12 radial sectors at 10 axial levels as previously described by *Kabaya et al*. [18], Fig. 2. The radial sector based Aortic Calcification Index (ACI) correlated closely with the volumetric calculation of aortic calcification (*r* =0.957, *p* < 0.001) and as both methods showed similar associations, for simplicity we therefore report results using the ACI method only.

The visceral and abdominal wall adipose assessment was performed using Osirix version 3.7.1. This involves analysing the fat content (visceral and subcutaneous) from a single slice, 6 cm above the L4/L5 disc using a previously validated and published method [15].

### Statistical considerations

Data was entered onto a dedicated study database and summarized using mean and standard deviations (sd) or medians and intra-quartile range (IQR) as appropriate for their distribution. Outlying and clinically implausible values were reconciled against the original clinical record. Correlations were examined using the non-parametric Spearman rank method. Hypothesis testing was performed using the Man Whitney *U* test. Analysis was conducted using IBM SPSS Statistics version 21 using a 2 sided type one error rate of 0.05.

## Results

The baseline clinical characteristics of the 64 study participants are shown in Table 1. Age ranged from 18.4 to 74.2 years. All subjects had additional risk factors for fragility fractures. Two subjects had undergone pre-emptive transplantation. The median (IQR) duration of End Stage Kidney Disease was 5.4 years (3.2 -10.0). Ten subjects (15.6 %) had diabetes; the attributed cause of ESKD in 6, while it developed post-transplantation in 4. Six subjects had a past history of atherosclerotic vascular disease, including 3 who had undergone a coronary revascularization procedure. The majority of subjects were immunosuppressed using Tacrolimus and Mycophenolate Mofetil, with over half having undergone successful post-transplant corticosteroid withdrawal. The percentage of participants with CKD stage 2 T, 3AT, and 3BT were 36 %, 30 %, 34 % respectively. The majority of subjects had residual post-transplant hyperparathyroidism, with a median iPTH of 91.5 ng/ml. Six subjects were treated with vitamin D supplementation and 7 with Bisphosphonates, of whom 6 meet WHO criteria for osteopenia and 1 for normal BMD on the basis of their study femoral hip iDXA scans.

**Table 1:** Patient clinical characteristics, medication use and laboratory parameters, overall and by the presence of abdominal calcification

	Total	ACI Score = 0	ACI Score >0	*p*-value
	(*n* = 64)	(*n* = 22)	(*n* = 42)	
Demographics & Clinical features				
Age (years), mean (sd)	47.3 (13.0)	35.8 (8.5)	52.3 (9.6)	0.001
Male: n (%)	39 (61 %)	18 (75 %)	21 (52 %)	0.07
Dialysis Modality:				0.95
Haemodialysis	39 (60.9 %)	15 (62.5 %)	24 (60 %)	
Peritoneal dialysis	23 (35.9 %)	8 (33.3 %)	15 (37.5 %)	
None (Pre-emptive)	2 (3.2 %)	1 (4.2 %)	1 (2.5 %)	
Duration of dialysis (years),	2.3	1.6	3.0	0.03
Median (IQR)	(1.6, 3.3)	(0.8, 2.0)	(1.7, 3.8)	
Duration of Transplant (years),	3.7	3.2	4.1	0.40
Median (IQR)	(0.9, 8.1)	(0.9, 8.8)	(0.9, 7.1)	
Mean Arterial BP (mm Hg), Mean (sd)	102.7 (9.7)	100.0 (8.8)	104.7 (8.9)	0.01
Body Mass Index (Kg/m^2^)	25.6	23.7	26.0	0.16
Median (IQR)	(22.8, 30.0)	(22.1, 29.3)	(23.4, 30.1)	
Diabetes: N (%)	10 (15.6 %)	3 (12.5 %)	7 (17.5 %)	0.60
Ever Smoked: N (%)	28 (43.8 %)	8 (33.3 %)	20 (50 %)	0.19
Prior parathyroidectomy N (%)	8 (12.5 %)	3 (12.5 %)	5 (12.5 %)	1.0
Past History CV event: N (%)	6	0	6	0.05
Current Medication Use				
Tacrolimus: N (%)	56 (87.5 %)	21 (87.5 %)	35 (87.5 %)	1.0
Mycophenolate mofetil: N (%)	52 (81.2 %)	17 (70.8 %)	35 (87.5 %)	0.10
Corticosteroid, N (%)	28 (43.8 %)	10 (41.7 %)	18 (45 %)	0.80
Vitamin D: N (%)	5	1	4	0.64
Laboratory parameters				
eGFR (ml/min per1.73 m^2^) mean (sd)	54.1 (17.6)	57.8 (14.8)	48.3 (14.9)	0.002
iPTH (ng/ml), median (IQR)	91.5 (73,108)	106.5 (83.5, 75.0)	86.5 (57,128)	0.85
Alkaline Phosphatase	106.2 (50.7)	106.5 (38.3)	106.0 (57.3)	0.97
Serum calcium (mmol/L), mean (sd)	2.57 (0.17)	2.52 (0.16)	2.63 (0.17)	0.06
Serum Phosphate (mmol/L), mean (sd)	1.01 (0.22)	1.00 (0.24)	1.02 (0.21)	0.70
Serum Cholesterol (mmol/L), Mean (sd)	4.7 (1.1)	4.2 (0.9)	5.0 (1.0)	0.006
Serum LDL Cholesterol (mmol/L), mean (sd)	2.65 (0.91)	2.34 (0.78)	2.82 (0.94)	0.06
Serum HDL Cholesterol (mmol/L), mean (sd)	1.35 (0.40)	1.28 (0.35)	1.39 (0.42)	0.36

### Bone mineral density

**Fig. 3:**
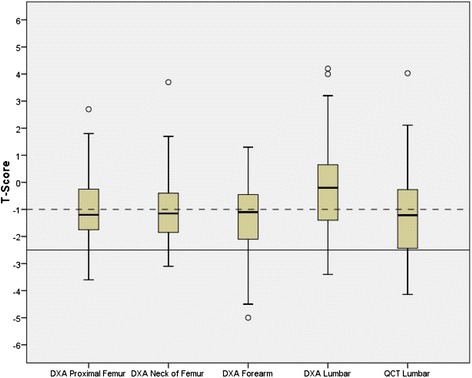
Boxplot showing median (solid line) and intra-quartile range (box) of IDXA measured areal Bone Mineral Density by site and QCT measured volumetric Bone Mineral Density at the lumbar spine

Measurements revealed a substantial degree of osteoporosis and osteopenia within the study population, Table 2. In keeping with the high prevalence of hyperparathyroidism, the BMD at the distal radius site - which is predominantly comprised of cortical bone and thus is especially susceptible to hyperparathyroidism - was substantially lower (663 g/cm^2^) than the proximal femoral site (946 g/cm^2^). Mean BMD at the lumbar site was 1163 g/cm^2^ by IDXA and only 139 g/cm^3^ by QCT. These patterns are reflected in the site specific T-scores measured by the 2 techniques, whereby the distribution of T scores for the IDXA lumbar spine was higher than that of the distal radius or femur or the distribution of T scores by QCT at the lumbar spine, Fig. 3. The proportion of subjects diagnosed with osteoporosis using the WHO reference standard of IDXA T score at the proximal femur was 7 %; with site specific osteoporosis rates of 9 % at lumbar spine and 17 % at the distal radius. The American College of Radiologists criteria which are based on volumetric BMD cutoffs classified similar number of subjects using QCT as by DXA scanning at the proximal femur as having underlying osteoporosis but fewer as having underlying osteopenia, Table 2.

**Table 2:** Areal and volumetric bone mineral density, T scores and diagnosis

	Bone Mineral Density		T-score		Diagnosis	
	Mean (sd)	r^a^	Median (IQR)	r^a^	% Osteo-porosis	% Osteo-paenia
DXA: Proximal femur	946 g/cm^2^ (179)	1.0 (ref)	−1.2 (−1.8, −0.3)	1.0 (ref)	7 %	53 %
DXA: Neck Of Femur	912 g/cm^2^ (168)	0.85, P < 0.001	−1.2 (−1.9,-0.4)	0.90	13 %	50 %
				P < 0.001		
DXA: Distal Radius	663 g/cm^2^ (119)	0.58, P < 0.001	−1.1 (−2.1,-0.5)	0.45	17 %	43 %
				P < 0.001		
DXA lumbar Spine	1163 g/cm^2^ (208)	0.73, p < 0.001	−0.2 (−1.4, 0.7)	0.70	9 %	28 %
				P < 0.001		
QCT: lumbar, Trabecular	139 g/cm^3^ (43)	0.68, P < 0.001	−1.2 (−2.4, −0.3)	0.61	9 %^b^	20%^b^
				P < 0.001		
QCT: Lumbar, Cortical	469 g/cm^3^ (32)	0.18, *P* = 0.17	n/a	n/a	n/a	n/a
QCT: Lumbar Total	606 g/cm^3^ (50)	0.65, *P* < 0.001	n/a	n/a	n/a	n/a

^a^ Correlation using Spearman rank method

^b^Using American College of Radiology cutoffs of QCT trabecular BMD <80 g/cm^3^ as being suggestive of osteoporosis and 80–120 g/cm^3^ as being suggestive of osteopenia

The Trabecular QCT volumetric BMD significantly correlated with areal BMD at the proximal femur (*r* = 0.68, *p* < 0.001), lumbar spine (*r* = 0.61, *p* < 0.001) and distal radius (*r* = 0.32, *p* = 0.02) but not with QCT measured cortical volumetric BMD (−0.06, *p* = 0.7). Cortical lumbar BMD only correlated significantly with areal BMD at the lumbar site (*r* =0.32, *p* = 0.01) but not at the distal radius (*r* =0.22, *p* = 0.1) or at other sites.

### Vascular calcification

**Fig. 4:**
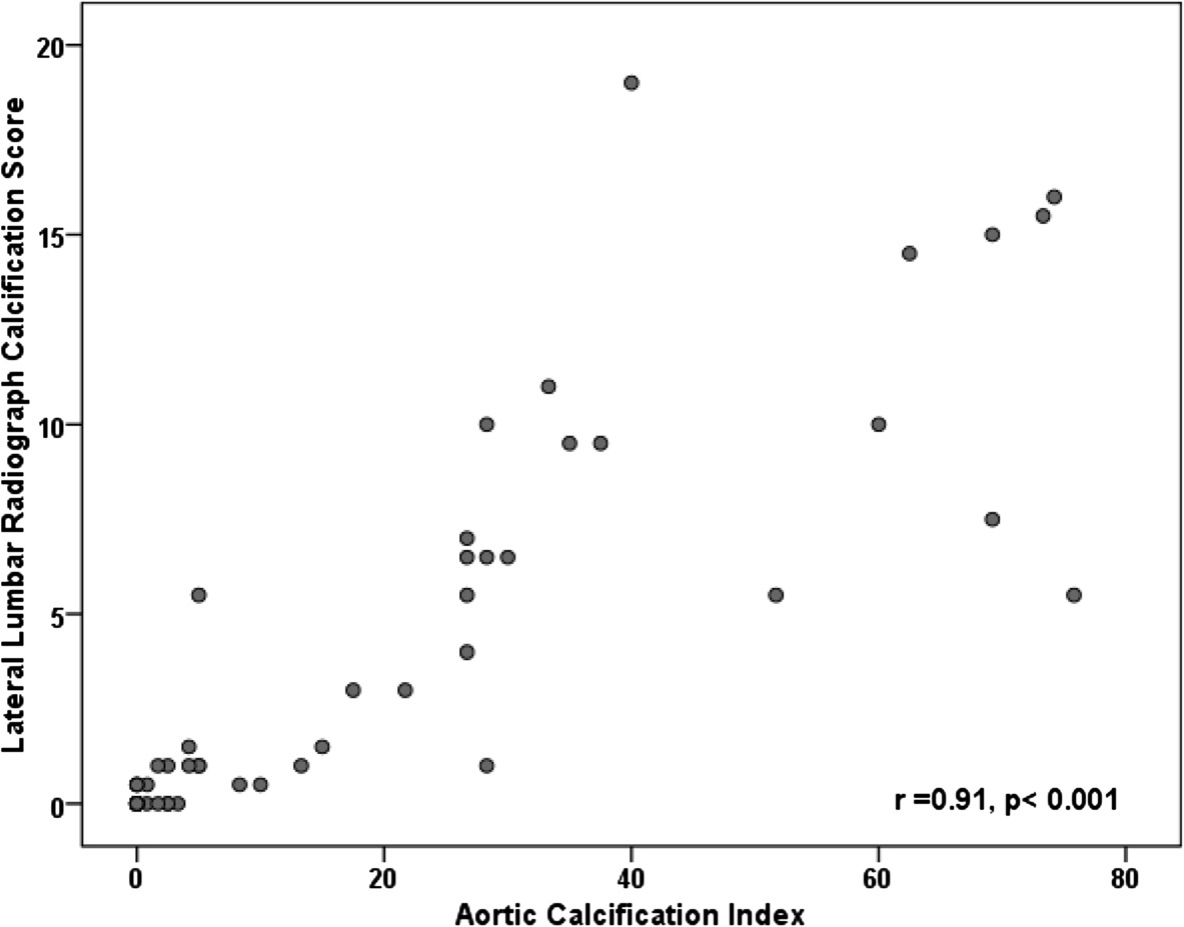
Scatterplot of the relationship between Lumbar Radiograph Measurement of Calcification and CT Determined Aortic Calcification Index

42 patients (64.6 %) had evidence of arterial calcification on CT. Not unexpectedly, the CT assessment identified more patients with aortic calcification than plain radiography appraisal, which identified 25 patients. Lumbar radiograph measurement of aortic calcification correlated closely with Aortic Calcification Index, (*r* = 0.91, *p* < 0.001, Fig. 4). The ACI differed significantly with duration of dialysis, with mean arterial pressure, hyperlipidaemia, a history of prior cardiovascular events and with post-transplant allograft function, Table 1. The ACI correlated with arterial Pulse Wave Velocity (Pulse Trace 400) (*r* = 0.29, *p* = 0.02) and pulse pressure (*r* = 0.51, *p* < 0.001). ACI was negatively correlated with Trabecular BMD (*r* = −0.31, *p* = 0.01) and positively with cortical volumetric BMD (*r* = 0.27, *p* = 0.03), Table 3.

**Table 3:** ᅟ

	Age (years)	eGFR	iPTH (ng/ml)	MAP (mmHg)	Pulse pressure	PWV (m/s)	ACI	X-ray calcification score	iDXA femoral T-score	QCT Trabecular	QCT Cortical
Age (years)	1	**−0.43 (** ***p*** **< 001)**	0.15 (*p* = 0.9)	**0.30 (** ***p*** **= 0.02)**	**0.41 (** ***p*** **< 001)**	0.19 (*p* = 0.14)	**0.72 (** ***p*** **< 001)**	**0.67 (** ***p*** **< 001)**	−0.17 (*p* = 0.2)	**−0.38 (** ***p*** **= 0.002)**	**0.34 (** ***p*** **= 0.006)**
eGFR (ml/min per 1.73 m^2)^)	**−0.43 (** ***p*** **< 0.001)**	1	−0.16 (*p* = 0.2)	**−0.34 (** ***p*** **< 0.01)**	**−0.37 (** ***p*** **< 0.01)**	0.09 (*p* = 0.5)	**−0.31 (** ***p*** **= 0.01)**	−0.25 (*p* = 0.06)	0.09 (*p* = 0.5)	0.09 (*p* = 0.5)	−0.12 (*p* = 0.3)
iPTH (ng/ml)	0.15 (*p* = 0.9)	−0.16 (*p* = 0.2)	1	0.07(*p* = 0.6)	−0.08 (*p* = 0.5)	0.01 (*p* = 0.9)	−0.01 (*p* = 0.9)	−0.12 (*p* = 0.4)	−0.04 (*p* = 0.8)	0.10 (*p* = 0.5)	**−0.29 (** ***p*** **= 0.02)**
MAP (mm Hg)	0.29 (*p* = 0.2)	−**0.34 (** ***p*** **< 0.01)**	0.07 (*p* = 0.6)	1	**0.59 (** ***p*** **< 0.001)**	0.07 (*p* = 0.6)	**0.42 (** ***p*** **< 0.001)**	**0.40 (** ***p*** **< 0.01)**	**−0.33 (** ***p*** **= 0.01)**	−0.21 (*p* = 0.09)	−0.16 (*p* = 0.2)
Pulse pressure (mm Hg)	**0.41 (** ***p*** **= 0.001)**	**−0.37 (** ***p*** **< 0.01)**	−0.08 (*p* = 0.5)	**0.59 (** ***p*** **< 0.001)**	1	0.02 (*p* = 0.9)	**0.51 (** ***p*** **< 0.001)**	**0.46 (** ***p*** **< 0.001)**	−0.16 (*p* = 0.2)	−0.15 (*p* = 0.3)	−0.02 (*p* = 0.9)
Pulse Wave Velocity (m/s)	0.19 (*p* = 0.14)	0.09 (*p* = 0.5)	0.01 (*p* = 0.9)	0.07 (*p* = 0.6)	0.02 (*p* = 0.9)	1	0.29 (*p* = 0.02)	0.28 (*p* = 0.04)	0.32 (*p* = 0.01)	0.17 (*p* = 0.2)	0.30 (*p* = 0.02)
ACI	**0.72 (** ***p*** **< 0.001)**	−0.31 **(** ***p*** **= 0.01)**	−0.01 **(** *p* = 0.9)	**0.42 (** ***p*** **= 0.001)**	**0.51 (** ***p*** **< 0.001)**	0.29 **(** ***p*** **< 0.02)**	1	0.91 **(** ***p*** **< 0.001)**	−0.19 (*p* = 0.15)	**−0.31 (** ***p*** **= 0.01)**	**0.27 (** ***p*** **= 0.03)**
X-ray calcification score	0.67 **(** ***p*** **< 0.001)**	−0.25 (*p* = 0.06)	−0.12 (*p* = 0.4)	**0.40 (** ***p*** **< 0.01)**	0.46 **(** ***p*** **< 0.001)**	**0.28 (** ***p*** **= 0.04)**	0.91 **(** ***p*** **< 0.001)**	1	−0.13 (*p* = 0.3)	−0.28 (*p* = 0.03)	0.19 (*p* = 0.15)
iDXA T-score Femoral head	−0.17 (*p* = 0.2)	0.09 (*p* = 0.5)	−0.04 (*p* = 0.8)	**−0.33 (** ***p*** **= 0.01)**	−0.16 (*p* = 0.2)	**0.32 (** ***p*** **= 0.01)**	−0.19 (*p* = 0.15)	−0.13 (*p* = 0.3)	1	**0.68 (** ***p*** **< 0.001)**	0.18 (*p* = 0.17)
QCT Trabecular BMD (g/cm^3^)	**−0.38 (** ***p*** **= 0.002)**	0.09 (*p* = 0.5)	0.10 (*p* = 0.5)	−0.21 (*p* = 0.09)	−0.15 (*p* = 0.3)	0.17 (*p* = 0.2)	−0.31 (*p* = 0.01)	−0.28 (*p* = 0.03)	0.68 (*p* < 0.001)	1	−0.07 (*p* = 0.6)
QCT Cortical BMD (g/cm^3^)	**0.34 (** ***p*** **= 0.006)**	−0.12 (*p* = 0.3)	**−0.29 (** ***p*** **= 0.02)**	−0.16 (*p* = 0.2)	−0.02 (*p* = 0.9)	0.30 (*p* = 0.02)	**0.27 (** ***p*** **= 0.03)**	0.19 (*p* = 0.15)	0.18(*p* = 0.17)	−0.07 (*p* = 0.6)	1

*Abbreviations*: *eGFR* estimated Glomerular Filtration Rate, *iPTH* intact Parathyroid Hormone, *MAP* Mean Arterial Pressure, *PWV* Pulse Wave velocity, *ACI* CT derived Aortic Calcification Index

### Fat mass

Body Mass Index Correlated with both Subcutaneous and Visceral Adipose Tissue volume, (*r* = 0.66 and *r* = 0.64 respectively, both *p* < 0.001). There was no significant relationship between total cholesterol and any of the CT measures of adiposity. Triglyceride level correlated with Visceral Adipose Tissue volume (VAT) (*r* = 0.43, *p* < 0.001), but not with Subcutaneous Adipose Tissue (SAT) volume. HDL level did not correlate with either VAT or SAT, however VAT did correlate negatively with LDL, although the relationship was weak, (*r* = −0.27, *p* = 0.05).

### Convenience

Acquisition time for each CT was equivalent to a standard CT lumbar spine with a patient time slot of 10 minutes each. The average time for analysis per patient was 3.5 minutes for BMD assessment (including cortical and trabecular), 2.1 minutes for fat appraisal and 5.6 min to complete both methods of calcium evaluation.

### Safety

The estimated effective radiation dose in this population was 2.4 mSv (range 2.0 – 2.7 mSv) per study, as calculated using the ImPACT CT Dosimetry tool (GE Healthcare). This compares to quoted effective radiation doses of 1.0 mSv for standard AP and lateral radiograph of lumbar spine [21].

## Discussion

The comprehensive ‘triple assessment’ of the evaluated patient cohort in this study using a single CT scan highlights the important clinical information that can be obtained in a manner that is efficient and convenient for both the patient and staff.

From the patient viewpoint it involves a single CT that takes less than ten minutes and which does not require preparation and does not involve contrast administration.

Fracture risk is elevated in Chronic Kidney Disease and End Stage Kidney Disease and the risk of fracture further increases post transplantation. The cumulative incidence of any fracture post transplantation is reported to be as high as 60 % [22] and is accompanied by an increased risk in all-cause mortality compared to fractures in the general population [23]. The pathogenesis of fragility fractures in CKD is more complicated than in the general population due to presence of underlying qualitative abnormalities of bone. This has resulted in recommendations against the routine use of bone density measurement in patients on dialysis, but with the restoration of endogenous renal function post transplantation current guidelines support the resumption of DXA screening.

The examination of spinal areal BMD by DXA is subject to several well recognized limitations, as the tradition posterior-anterior approach of DXA scanning cannot distinguish between calcification within the vertebrae and the extra-osseous structures such as osteophytes, osteochondrosis or –of special relevance in CKD- within the calcified wall of the abdominal aorta. Although the vertebral body is predominantly comprised of trabecular bone, the DXA measurement is a composite of both vertebral cortical and trabecular bone and is heavily influenced by the calcium content of the cortical bone that comprises the posterior elements of the vertebrae which contributes relatively little to the integrity of the vertebral body. Thus the DXA derived areal BMD lumbar T score is typically substantially higher than that measured at other sites as is also evident from our results as shown in Fig. 3. QCT by measuring a true volumetric BMD of the trabecular bone specific at the site at which the vertebral collapse occurs is able to avoid these limitations. The calculation of a QCT derived lumbar trabecular T score helps highlight the often extensive reduction in vertebral BMD as compared to the BMD of gender changed young adults and allows for inter-subject comparisons. This score however is not representative of the T-score that would be obtained using either a QCT or DXA at the proximal femur, as is the reference standard for the WHO diagnosis of osteoporosis and which is used in the FRAX calculation of long term fracture risk. Instead The American College of Radiology recommends cutoffs based on reduced volumetric BMD that are intended to replicate the diagnosis of underlying osteoporosis and osteopenia as would be obtained using the reference standard of femoral BMD. In our analysis the application of these cutoff resulted in similar identification of subjects with osteoporosis but the identification of substantially fewer subjects with osteopenia. The FDA has recently approved additional novel techniques in QCT that allow for the direct measurement of femoral areal BMD as is used as the reference standard in the diagnosis of osteoporosis and which can be obtained at the same time as measuring lumbar volumetric BMD.

The separate measurement of cortical and trabecular volumetric BMD is of special relevance in renal allograft recipients with persistent or progressive residual hyperparathyroidism, as this preferentially induces cortical bone loss, the impact of which can be seen in the measured areal BMD at the distal radius which is almost exclusively comprised of cortical bone and which demonstrates the lowest T score of all sites examined in our study and which moreover significantly correlates with the post-transplant iPTH level. In addition, as trabecular bone is more metabolically active than cortical bone, it responds more rapidly to either a change in bone turnover or to therapeutic interventions. Thus the specific measurement of trabecular volumetric BMD as well as indicating the risk of spinal collapse fracture and facilitating the diagnosis of osteoporosis, allows for more rapid assessment of the impact of a change in treatment or clinical circumstance. The fact that the two different forms of bone differ in their susceptibility and response to insults, and as in our study there was no correlation between QCT measured trabecular and cortical bone mineral density further underscores the rationale for their separate evaluation rather than the use of a composite measure. While the optimal treatment of osteoporosis post transplantation is unclear, accurate quantification of the severity of the bone mineral deficit and its change over time should inform any discussion of the potential risks and benefits of any proposed intervention. Furthermore the reliable early identification of decreased bone mineral density at least facilitates secondary risk reduction such as the selective application of corticosteroid minimization or avoidance; strategies that are beneficial to the bone but which are not devoid of risk to the allograft.

Vascular calcification is proposed to be a major driving force in increased cardiac afterload and heart failure in ESKD populations. Plain radiography is a useful non-invasive and inexpensive tool for detecting the presence of vascular calcification, both in the general population and in CKD. Kauppila at al introduced a semi-quantitative scoring system for vascular calcification using lateral radiographs of the lumbar spine. This scoring system was used in a study of 515 dialysis patients which showed the presence of aortic calcification to be predictive of all cause and cardiovascular mortality [24]. Carotid – Femoral (Aortic) Pulse Wave Velocity measures the speed with which the arterial pulse travels down the aorta and thus is a functional measure of aortic stiffness. It is inexpensive to perform and an independent predictor of cardiovascular mortality, [25] including in ESKD [26].

The technique is however very user dependent and subject to considerable inter-individual variability. The accurate assessment of vascular calcification should support aggressive cardiovascular risk reduction as well as potentially supporting prospective monitoring of cardiac function, while in a research setting it should facilitate a better understanding of the natural history and pathophysiology of vascular calcification post transplantation.

The pathogenic consequences of alterations in visceral fat mass post transplant is unclear and is a subject of substantial ongoing research. One limitation to exploring these relationships is the difficulty in accurately measuring visceral fat mass using routine clincal measures. While fat mass correlates with hip-waste circumference, such measures are often inaccurate and lack precision. The assessment of fat mass using single slice CT has been validated [14] and has been shown to be associated with the metabolic syndrome [27, 28] and increased cardiovascular risk [29]. Thus fat mass assessment as part of the triple assessment may further enhance cardiovascular risk factor stratification and should provide considerable research opportunitiues in the evaluation of this pathophysiological process.

Increasing attention has focused in recent times on the potential deleterious effects of cumulative radiation exposure. We were able to undertake the CT triple assessment using a protocol which imparts a radiation exposure approximately 2.5 times that of the standard lateral lumbosacral x-ray that is recommended by KDIGO . The increased precision and accuracy of CT assessment may result in a reduced number of studies especially in those patients who can be reliably shown to be free of calcification post engraftment. This incremental radiation exposure is further offset if the analysis is conducted as part of a CT scan that is otherwise scheduled for clinical reasons.

The potential cost implications of the proposed CT evaluation depends heavily on the local medical infrastructure and reimbursement environment. The application requires the presence of an appropriate CT service with sufficient scheduling flexibility as to allow for the additional study time of undertaking the QCT. The costs of the specific QCT software package and phantom for calculation of volumetric BMD would be expected to be less than that of the purchase of an entry level DXA scanner - assuming the presence of an existing CT service. From a staff viewpoint the time commitment to perform either the CT scan or the iDXA are similar, as are the approximate time requirements for reading the scans. Of note in Australia the measurement of volumetric BMD by QCT is reimbursed at the same rate as that of areal BMD by DXA. The CT has the additional cost effective element of providing additional information on the intra-abdominal structures avoiding the need for separately conducted imaging studies.

Limitations of our study are its limited sample size and that it is based in a single Irish institution. Within the current study we did not examine the inter or intra individual reproducibility of the techniques used, however, at least in the case of the assessment of aortic calcification the proposed technique would be expected to be less subjective than the existing approach using lateral lumbar x-rays. Additionally, due to the cross-sectional nature of our analysis, the accuracy of CT based measures of Bone Mineral Density, aortic calcification and adiposity in predicting fracture occurrence and cardiovascular events in the renal transplant population could not be determined. Follow-up of these outcomes over time may help clarify these issues.

## Conclusion

We demonstrate the feasibility of conducting accurate simultaneous assessment of lumbar Bone Mineral Density, aortic calcification and visceral fat mass using a specifically designed single CT protocol. This single CT scan offers a “one stop shop” approach to measuring these important indices. The resulting data is likely to be considerably more accurate than that obtained by methods currently in routine clinical use. The precise role and long term attributable benefit of this technique requires confirmation. However the above approach may be of substantial utility in a research setting and should be considered at least where repeated radiographic assessment of vascular calcification is planned as part of routine clinical practice.

## Abbreviations

ACI: Aortic calcification index
AP: Anteroposterior
BMD: Bone mineral density
BMI: Body mass index
CKD: Chronic kidney disease
CT: Computed tomography
DXA: Dual energy X-ray absorptiometry
EBCT: Electron beam computed tomography
ESKD: End stage kidney disease
HDL: High density lipoprotein
KDIGO: Kidney disease improving global outcomes
LDL: Low density lipoprotein
mSv: milliSievert
NODAT: New onset diabetes after transplantation
pQCT: Peripheral quantitative computed tomography
PWV: Pulse wave velocity
QCT: Quantitative computed tomography
SAT: Subcutaneous adipose tissue
SPSS: Statistical package for the social sciences
VAT: Visceral adipose tissue
